# Proteasome inhibition blocks necroptosis by attenuating death complex aggregation

**DOI:** 10.1038/s41419-018-0371-x

**Published:** 2018-03-01

**Authors:** Mohammad Ali, Edward S. Mocarski

**Affiliations:** Department of Microbiology & Immunology, Emory Vaccine Center, Emory University School of Medicine, 1462 Clifton Rd., Atlanta, GA 30322 USA

## Abstract

Proteasome inhibitors have achieved clinical success because they trigger intrinsic and extrinsic cell death to eliminate susceptible human cancers. The ubiquitin-proteasome protein degradation system regulates signaling pathways by controlling levels of components such as cellular inhibitor of apoptosis (cIAP)1 and cIAP2 in TNF-mediated cell death. Here, we sought to evaluate the contribution of necroptosis to the cell death pattern induced by the specific proteasome inhibitor Carfilzomib (Cf). Proteasome inhibitor-sensitive multiple myeloma cell lines die in response to Cf by apoptosis in combination with serine protease-dependent death, without any contribution of RIPK3-dependent necroptosis. Proteasome inhibition leads to the induction of apoptotic markers such as activated caspase-3 rather than necroptotic markers such as phosphorylated-MLKL in all cell lines tested. In HT-29 cells, Cf attenuates the late RIPK1 interaction with TNFR1 during TNF-induced necroptosis without altering the sensitivity of cIAP antagonists. Cf treatment results in decreased translocation of death signaling components RIPK1, FADD, caspase-8, cFLIP, and RIPK3 to detergent insoluble fractions. Our results show that proteasome inhibition with Cf impairs necroptosis and favors apoptosis even in cells with intact necroptotic machinery. Following the induction of TNFR1-mediated necroptosis, proteasome activity stabilizes effective aggregation and activation of ripoptosome/necrosome complexes.

## Introduction

The ubiquitin (Ub)-proteasome degradation system regulates the levels of proteins involved in receptor signaling pathways, such as those controlling cell death and cell cycle^1–3^. Notably, proteasome inhibition kills many human cancer cell lines and provides a strategy for therapeutic intervention in multiple myeloma (MM) as well as mantel cell carcinoma^3^. In general, proteasome inhibition results in the accumulation of misfolded and polyubiquitinated proteins that activate the terminal ER stress response leading to mitochondrial release of cytochrome *c* and serine proteases^4^. In addition, proteasome inhibition triggers TRAIL-dependent apoptosis in some human cancer cell lines^5^. In contrast to observations in human cells, proteasome inhibition induces RIPK3-dependent necroptosis of mouse fibroblasts associated with accumulation of polyubiquitinated RIPK3^6^. In either mouse or human cells, proteasome inhibition has been shown to block NFκB activation by stabilizing IκBα^3^, attenuating the TNF-mediated survival response.

Necroptosis is a form of regulated lytic cell death characterized by swelling of intracellular organelles and leakage through the plasma membrane^7^ triggered by TNF family death ligands^8^, pathogen recognition^9^, T cell activation^10^ interferon^11^ or virus infection^12,13^ particularly when caspase activation is compromised. This pathway contributes to host defense during infection^14–16^ as well as to inflammatory tissue injury^12,17,18^. Considerable understanding of necroptosis stems from studies of TNF receptor (TNFR) 1 signaling. TNFR1 activation leads to the recruitment of an Ub ligation complex that includes the TNFR-associated factor (TRAF)2 and the cellular inhibitor of apoptosis (cIAP)1 and cIAP2. This complex adds K63-linked Ub chains to TNFR1 associated signaling components including receptor interacting protein (RIPK)1^7^, favoring the activation of the NFκB survival pathway^19–21^. It is therefore necessary to compromise NFκB function to favor TNFR1-induced death outcomes, either by blocking de novo protein synthesis^22^ or by compromising cIAP1 and cIAP2 using antagonists^23^ that mimic the natural impact of second mitochondria activator of caspases (SMAC). These undermine NFκB signaling and sensitize to cell death^24^ by inducing auto-ubiquitination and proteasomal degradation of cIAP1 and cIAP2^25–27^. Because SMAC mimetics stimulate degradation of cIAPs downstream of TNFR1 and toll-like receptor 3 (TLR3)^28^, as well as following genotoxic stress^29^, proteasome inhibitors would be predicted to counteract this degradation, preventing TNF-induced necroptosis and favoring survival.

Here we explore the impact of proteasome inhibition in human cancer cell lines. In contrast to the reported response of mouse fibroblasts^6^, both multiple myeloma (MM) cells and necroptosis-sensitive HT-29 adenocarcinoma cells favor apoptosis when treated with the highly specific proteasome inhibitor Carfilzomib (Cf). In MM cells, Cf drives caspase and serine protease combined death pathways. Moreover, in HT-29 necroptosis-sensitive cells, proteasome inhibition prevents activation of TNFR1-induced necroptosis and reduces ripoptosome^28^ and necrosome^30^ aggregation, as well as accumulation of phosphorylated mixed lineage kinase domain-like (MLKL) pseudokinase. Thus, proteasome inhibition blocks TNFR1-induced necroptosis independent of cIAP stability. Despite the overall pro-apoptotic impact of proteasome inhibitors on cancer cells, necroptosis is suppressed by Cf. Our findings define a checkpoint dependent on the Ub-proteasome system (UPS) during necroptosis execution.

## Results

### Cf fails to activate necroptosis in human cells

**Fig. 1:**
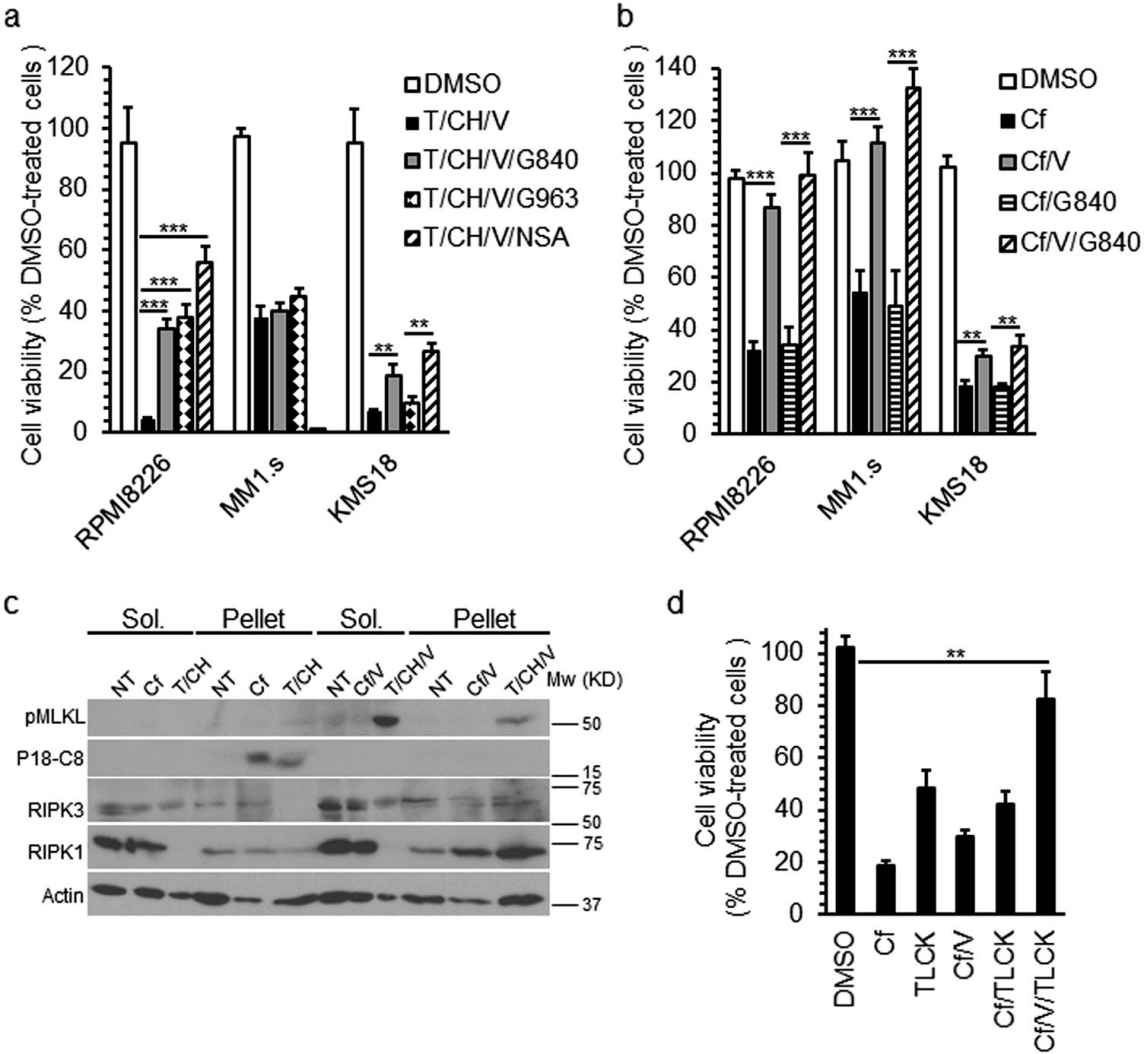
Evaluation of multiple myeloma (MM) cell line responses to Cf treatment. **a** Viability of indicated MM cell lines 22 h post treatment (hpt) with TNF + cycloheximide + *zVADfmk* (T/CH/V) measured by CellTiterGlow assay of four replicate wells per data point, either alone or in combination with 3 µM RIPK3 inhibitor GSK’840 (G840), or 3 µM RIPK1 inhibitor GSK’963 (G963), or 10 µM human MLKL-specific inhibitor necrosulfonamide (NSA). *p_*v*_ < 0.05, **p_*v*_ < 0.005, ***p_*v*_ < 0.0005. **b** Viability of MM cells 22 hpt with Cf either alone, or in combination with V, G840 or both together. **c** Immunoblot (IB) for phospho-MLKL (p)MLKL and Casp8 cleavage product P18 (P18-C8) in 1% Triton-soluble (Sol.) and -insoluble (Pellet) fractions of RPMI8226 cells 8 hpt with Cf, Cf/V, T/CH or T/CH/V. β-actin is used as a loading control. **d** Viability of KMS-18 cells 22 hpt with Cf alone, Cf/V, or in combination with the pan serine protease-inhibitor TLCK as indicated

The MM cell lines RPMI8226, MM1.s and KMS-18 are all killed by proteasome inhibitors^31^. Susceptibility of these cell lines to TNF-induced necroptosis was evaluated. Treatment with TNF (T), cycloheximide (CH) and zVAD*-fmk* (V) resulted in the induction of death in all three cell lines (Fig. 1a), showing susceptibility to caspase-independent death. RIPK3 inhibitor GSK'840 (G840), RIPK1 inhibitor GSK'963 (G963), or MLKL inhibitor necrosulfonamide (NSA) enhanced viability of RPMI8226 cells to T/CH/V, indicating a potential contribution of necroptosis^32^. Both G840 and NSA modestly improved KMS-18 cell viability, but G963 had no effect. G840 and G963 failed to improve MM1.s cell viability, and NSA was toxic. All three MM cell lines expressed comparable levels of RIPK1 (Supplementary Figure 1c). MLKL levels were equivalent in RPMI8226 and KMS18, but were lower in MM1.s, but RIPK3 was readily detectable only in RPMI8226 cells. When we treated the MM cells with Cf all three lines showed the expected^31^ sensitivity to Cf with a calculated IC_50_ ranging from 5 to 10 ηM (Supplementary Figure 1a). Caspase inhibition restored the viability of Cf-treated RPMI8226 and MM.1s cells, but RIPK3 inhibition had no impact even in RIPK3-expressing RPMI8226 cells (Fig. 1b). Treatment with Cf or Cf/V failed to induce pMLKL even though RPMI8226 cells showed induction following T/CH/V (Supplementary Figure 1b) along with translocation of pMLKL to the detergent-insoluble pellet (Fig. 1c), a step that precedes cell leakage^33^. Cf treatment induced processing of Casp8 (P55-C8 vs. P42-C8 and P18-C8) and Casp3 (Cl-C3) that was inhibited by V (Fig. 1c and Supplementary Figure 1b) in a pattern consistent with apoptosis. Although cell death proteins RIPK1, RIPK3, Casp8, and MLKL all translocated to the pellet fraction of RPMI8226 cells following T/CH/V^14,33,34^, treatment with Cf alone or Cf/V did not drive pMLKL into the pellet fractions (Fig. 1c). Instead, fully active P18-C8 translocated to the pellet in levels comparable to those occurring in T/CH-induced apoptosis (Fig. 1c). KMS-18 cells remained largely susceptible to Cf-induced death despite the inhibition of caspases as well as RIPK3 kinase activity (Fig. 1b). Given that Casp8 and Casp3 processing was inhibited by V (Supplementary Figure 1b) additional pathways likely contributed to death such as those dependent on release of serine proteases from mitochondria^4^. The pan-serine protease inhibitor TLCK was toxic to KMS-18 cells, although in combination with V inhibited this death (Fig. 1d). Thus, Cf induces combined death pathways in MM cells, independent of RIPK3 kinase function.

**Fig. 2:**
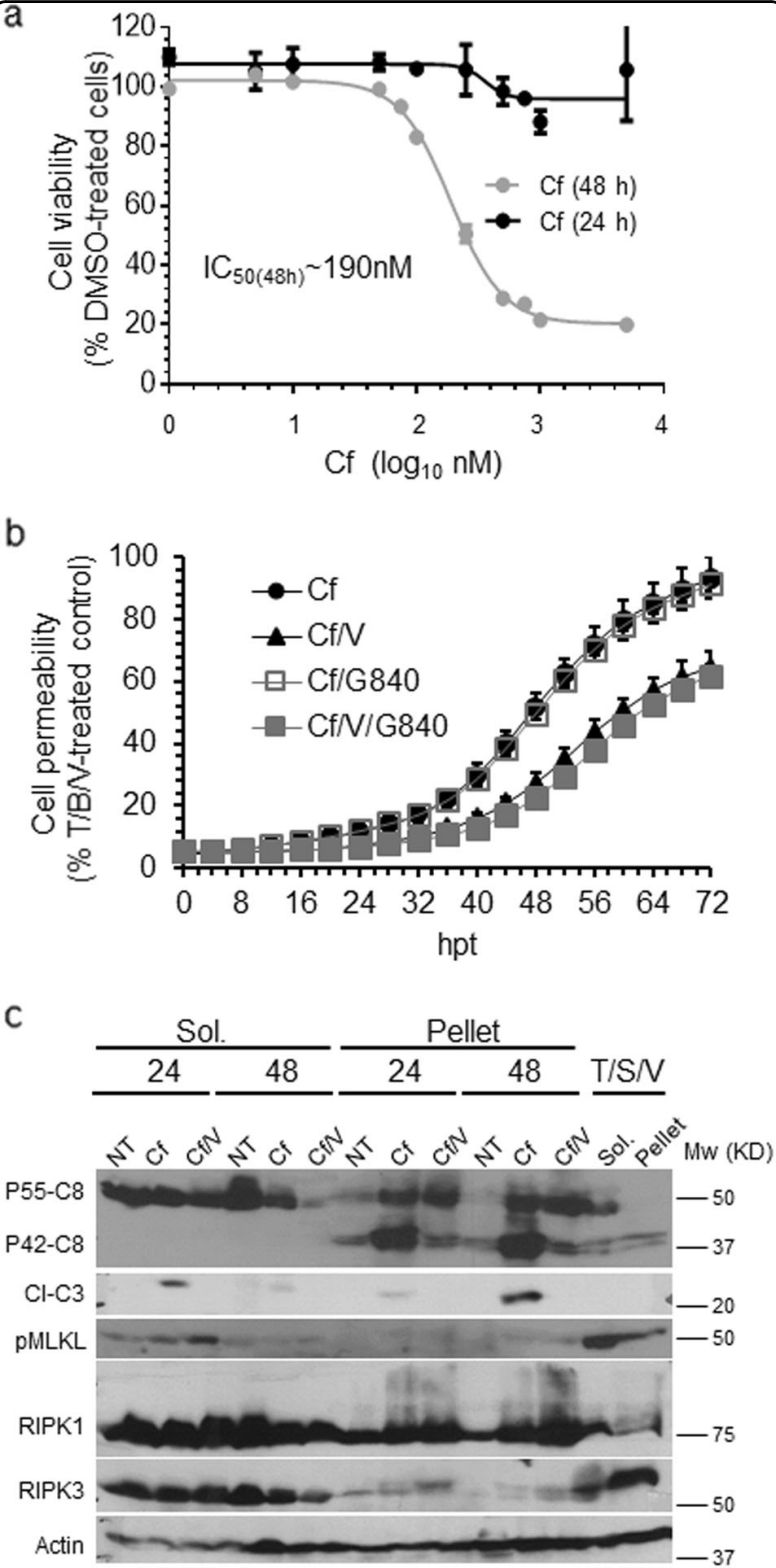
Evaluation of HT-29 colon carcinoma cells response to Cf treatment. **a** HT-29 cell viability following treatment with indicated concentrations of Cf for 24 h (black line) or 48 h (gray line). **b** Time course of HT-29 cell membrane permeability following treatment with Cf alone, Cf/V, Cf/G840 or Cf/V/G840 depicted as a percentage of T/S/V-treated control. **c** IB of Casp8 (P55-C8 and P42-C8), cleaved Casp3 (Cl-C3), pMLKL, total RIPK1, and total RIPK3, in Sol. and Pellet HT-29 cell fractions either not treated (NT) or treated with Cf or Cf/V for the indicated time with control T/S/V (6 hpt) cell fractions

We next evaluated proteasome inhibitor-induced death in necroptosis-sensitive HT-29 cells. These cells resisted Cf toxicity such that plasma membrane permeability was delayed until 40 h post treatment (hpt) (Fig. 2a, b). Caspase inhibition with V attenuated death independent of RIPK3 kinase inhibition. Treatment with Cf for at least 24 h resulted in cleavage of Casp8 and Casp3 in soluble and pellet fractions (Fig. 2c) consistent with apoptosis, despite the slight induction of pMLKL by Cf or Cf/V. Thus, necroptosis does not contribute to proteasome inhibitor-induced death in cells that have provided key insights into alternate apoptotic and necroptotic pathways^8^.

### Cf inhibits TNF receptor-induced necroptosis

**Fig. 3:**
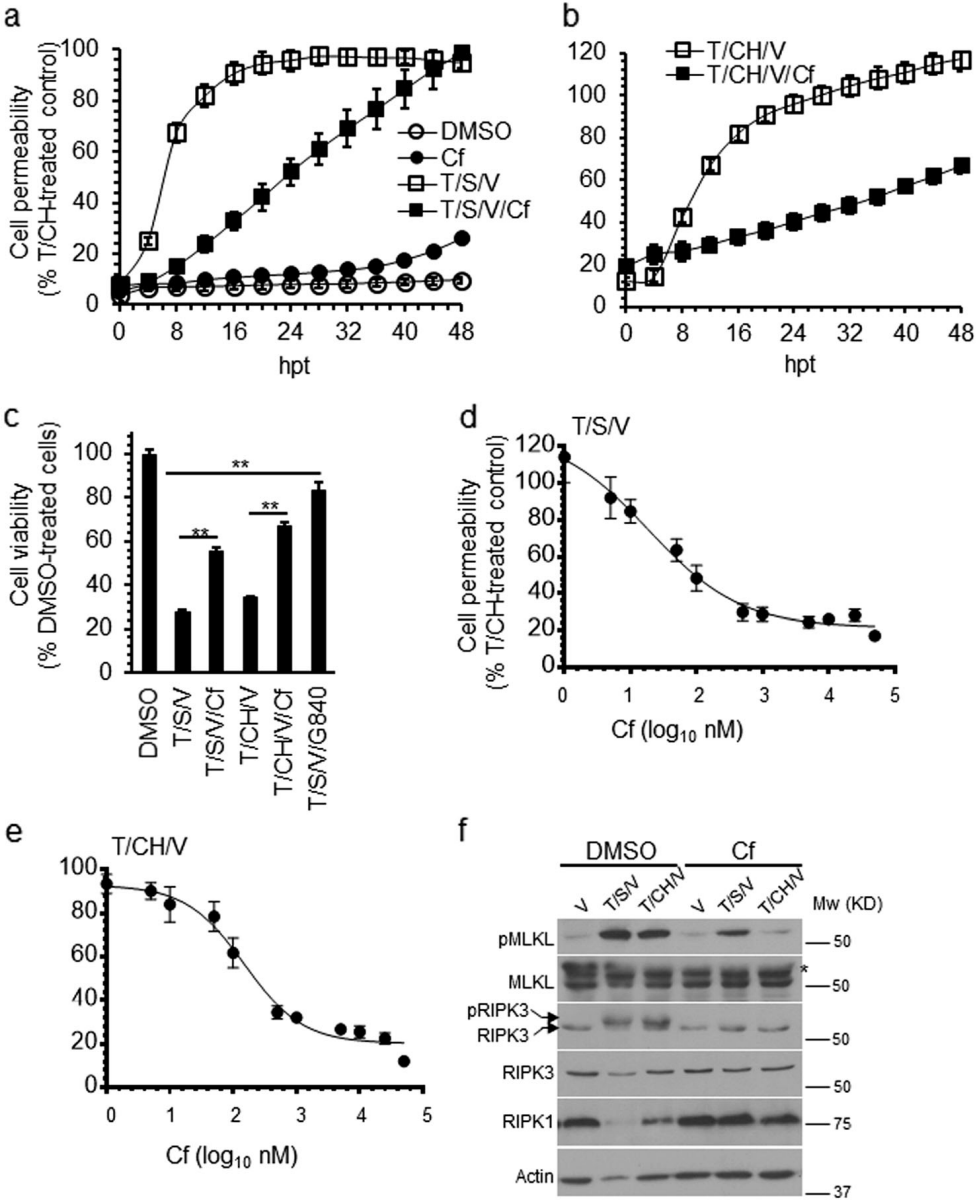
Attenuation of TNFR1-induced necroptosis by Cf. **a**, **b** Time course of HT-29 cell membrane permeability following treatment with Cf, T/S/V or T/S/V/Cf (**a**) or with T/CH/V or T/CH/V/Cf (**b**), depicted as a percentage of T/CH-treated (24 h) control. **c** Viability of HT-29 cells 24 hpt under the indicated conditions. **d**, **e** Membrane permeability in HT-29 cells 24 hpt with the indicated concentrations of Cf following treatment with T/S/V (**d**) or T/CH/V (**e**). **f** IB of total cell lysates collected 6 hpt for pMLKL, MLKL (with asterisks indicating cross-reactive bands), pRIPK3, RIPK3, and RIPK1 under the indicated conditions

We next evaluated the impact of Cf on the necroptosis in HT-29 cells. The response to either T, SMAC mimetic, and V (T/S/V) or T/CH/V was attenuated by Cf (Fig. 3a–c) in a dose-dependent manner (Fig. 3d, e). Cf was sufficient to inhibit T/S/V or T/CH/V induction of pRIPK3 and pMLKL and stabilize RIPK1 and RIPK3 levels (Fig. 3f) at 8 hpt. Cf also decreased the levels of T/CH/V-induced pMLKL in RPMI8226 cells (Supplementary Figure 2a) and rescued T/CH/V-induced membrane permeability in HaCaT cells (Supplementary Figure 2 b). Moreover, the commonly used proteasome inhibitor MG132 also protected HT-29 cells from TNFR1-induced necroptosis (Supplementary Figure 2c and d). These results reveal an unexpected requirement for proteasome activity during TNFR1-induced necroptosis.

### Cf inhibits necroptosis independent of cIAP1 and cIAP2 stability

**Fig. 4:**
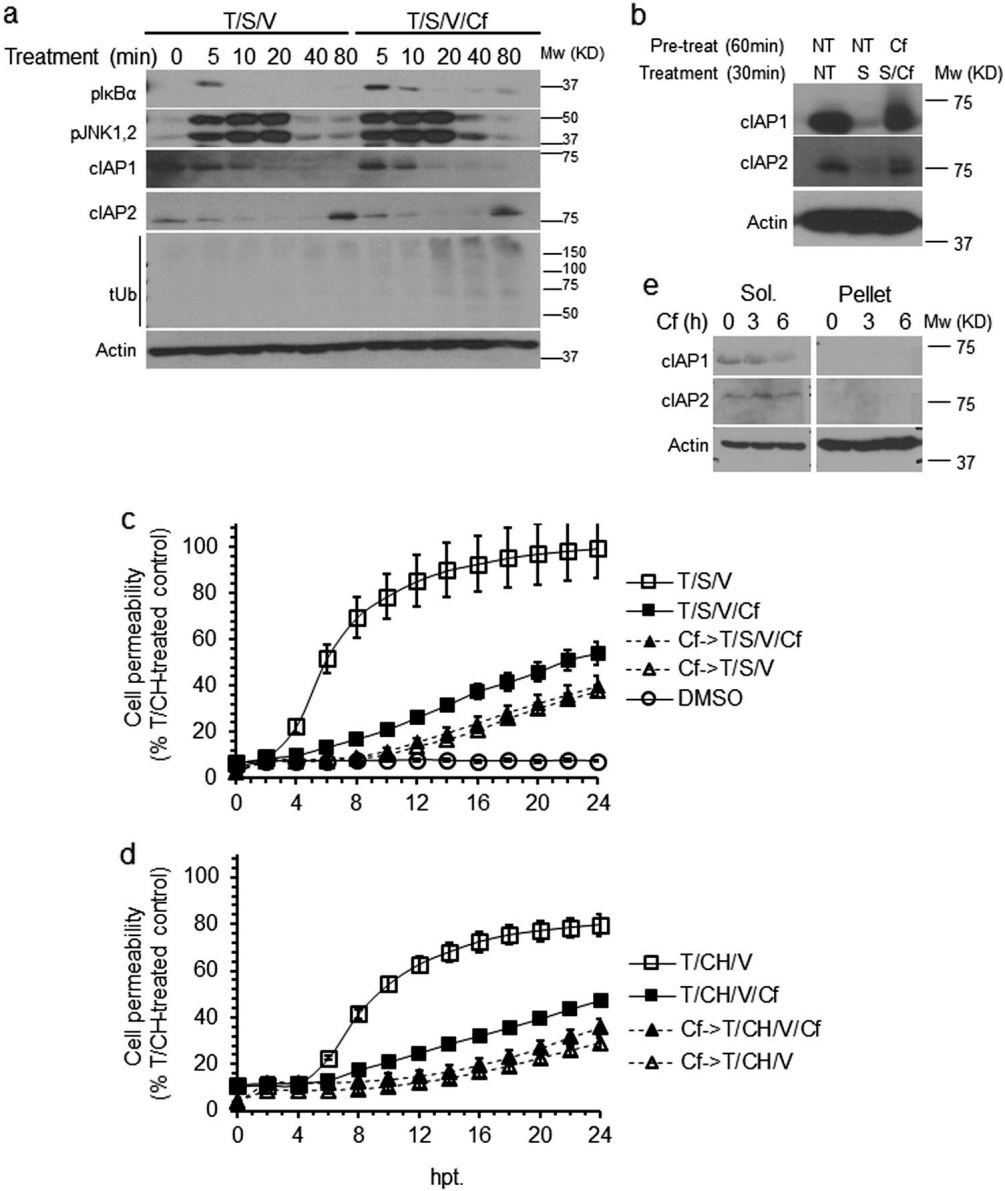
Impact of Cf on necroptosis signaling. **a** IB of pIκBα, pJNK1/pJNK2, cIAP1, cIAP2 and total Ub (tUb) in HT-29 cells treated with T/S/V or with T/S/V/Cf for the indicated times. Size marker is shown to the left of tUB blot. **b** IB of cIAP1 and cIAP2 in HT-29 cells either NT or pretreated with Cf for 60 min followed by treatment with S or S/Cf for 30 min. **c**, **d** Time course of membrane permeability in HT-29 cells either NT or pretreated with Cf followed by treatment with T/S/V (Cf- > T/S/V) or T/S/V/Cf (Cf- > T/S/V/Cf) (**c**) or with T/CH/V (Cf- > T/CH/V) or T/CH/V/Cf (Cf- > T/CH/V/Cf) (**d**), depicted as a percentage of T/CH (24 hpt) control. **e** IB of cIAP1 and cIAP2 in HT-29 cells treated with Cf for the indicated time

To investigate the ability of proteasome inhibitors to antagonize SMAC mimetic-induced degradation of cIAP1 and cIAP2^24,35^, we first tested the effect of Cf on TNFR1 complex I-dependent signaling in HT-29 cells. T/S/V resulted in the expected^36^ transient phosphorylation of IκBα (pIκBα) and JNK1/JNK2 (pJNK1/2), along with rapid depletion of cIAP1 and cIAP2 (Fig. 4a). Cf stabilized pIκBα levels in a pattern consistent with inhibition of proteasome function^37^, without much impact on pJNK1/2 levels. Cf had little impact on cIAP1 and cIAP2 degradation, but promoted accumulation of total ubiquitinated proteins after only 20 min of treatment (Fig. 4a). Pretreatment with Cf for 60 min prevented S-dependent degradation of cIAPs (Fig. 4b), consistent with a contribution of the proteasome to cIAP regulation^35^. These results indicate that Cf blocks TNFR1-induced necroptosis independent of an impact on cIAP degradation (Fig. 4c, d). Notably, Cf did not increase the levels of cIAPs, but appeared to reduce the levels of cIAP1 (Fig. 4e). Thus, proteasome activity sustains necroptosis in a manner independent cIAP fate.

**Fig. 5:**
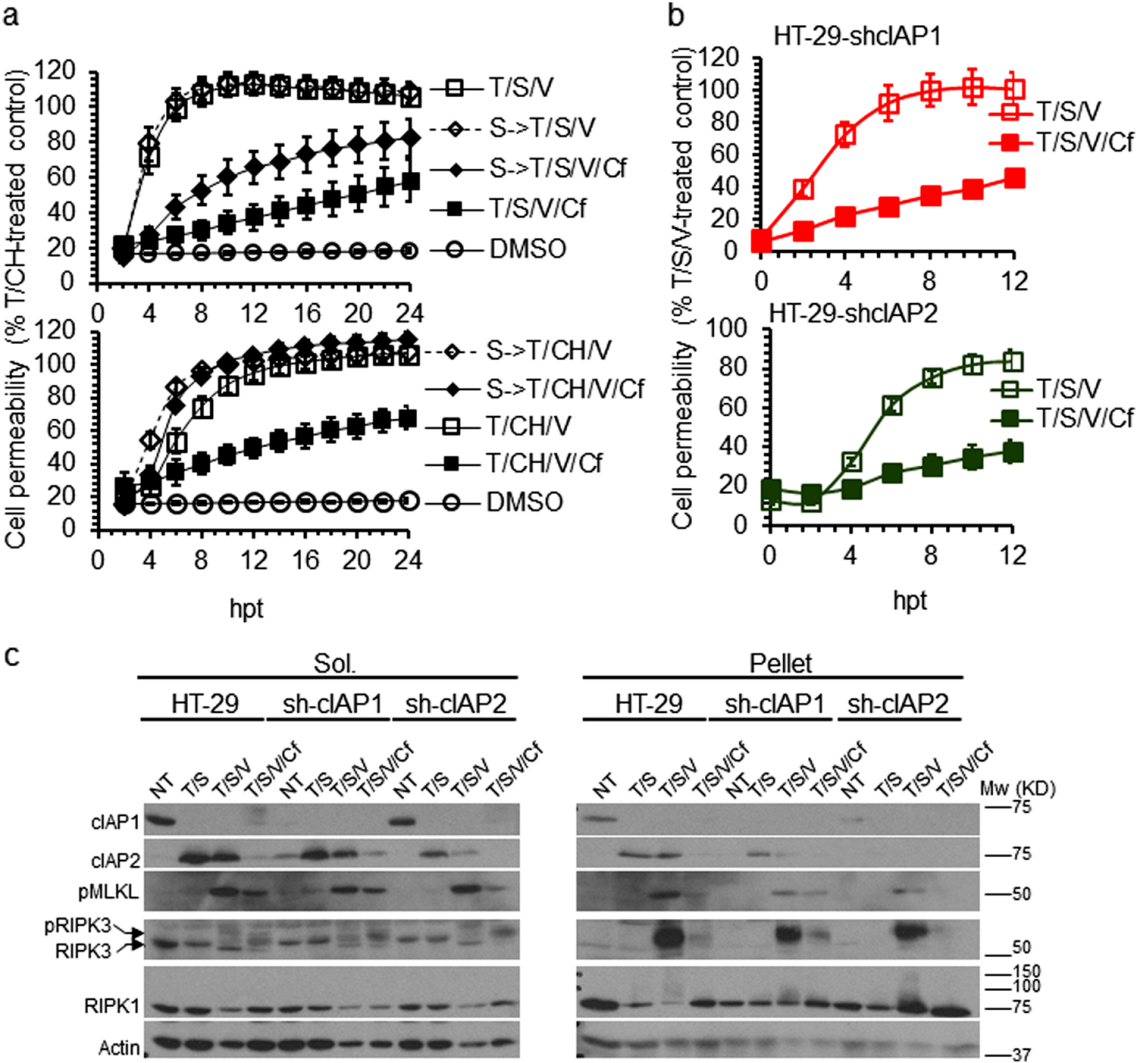
Cf effect on TNFR1-induced necroptosis in cIAP-depleted cells. **a** Time course of membrane permeability in HT-29 cells either NT or pretreated with S followed by treatment with T/S/V (S- > T/S/V) or T/S/V/Cf (S- > T/S/V/Cf) (upper panel) or with T/CH/V (S- > T/CH/V) or T/CH/V/Cf (S- > T/CH/V/Cf) (lower panel), depicted as a percentage of T/CH (24 hpt) control. **b** Time course of cell membrane permeability of HT-29-shcIAP1 (upper panel) and HT-29-shcIAP2 (lower panel) following treatment with T/S/V or T/S/V/Cf. **c** IB of cIAP1, cIAP2, RIPK1, RIPK3, and pMLKL in Sol. and Pellet cell fractions of HT-29 cells, HT-29-shcIAP1, and HT-29-shcIAP2 cells following 6 hpt with T/S, T/S/V, or T/S/V/Cf, as indicated

Following T/S/V treatment for 80 min, cIAP2 became elevated even when Cf was present (Fig. 4a). To determine whether this increase in cIAP2^38^ inhibited death, we pretreated HT-29 cells with S for 30 min to deplete cIAPs prior to cell death induction (S- > T/S/V or S- > T/CH/V) without altering the pattern of necroptosis. Cf attenuated cell permeability induction by T/S/V but not by T/CH/V (Fig. 5a), indicating a role for protein synthesis in Cf protective effect. T/S/V/Cf resulted in complete elimination of cIAP1 without affecting cIAP2 levels. T/CH/V/Cf did not affect cIAP1 levels but led to a massive elevation of cIAP2 (Supplementary Figure 3). S pretreatment eliminated cIAP1, reduced the levels of cIAP2 in T/S/V/Cf-treated cells, and eliminated cIAP2 in T/CH/V/Cf treated cells. These patterns reinforce an impact of Cf on stability of de novo synthesized cIAP2 during necroptosis, independent of cIAP1^38^.

To investigate the contribution of cIAP2 to Cf-mediated cell death suppression, we generated HT-29 cells with stable knockdown of cIAP1 and cIAP2. Out of the four sh-HT-29 cell lines generated, sh-cIAP1a and sh-cIAP2b (here referred as sh-cIAP1 and sh-cIAP2) showed the expected pattern, proliferating as well as parental HT-29 cells (Supplementary Figure 4a and data not shown). Knockdown of cIAP1 or cIAP2 did not increase sensitivity to Cf or T/S/V (Supplementary Figure 4b and c), and did not alter the pattern of inhibition by Cf (Fig. 5b). T/S or T/S/V eliminated cIAP1 but drove induction and translocation of cIAP2 along with pRIPK3 and pMLKL, along with modified RIPK1, to the pellet fraction (Fig. 5c). Thus, cIAP knockdown did not alter either apoptosis or necroptosis. Cf did not alter cIAP1 degradation, but was associated with depletion of cIAP2, reduced induction and translocation of pMLKL and pRIPK3, and reduced RIPK1 modification in the pellet fraction. Cf therefore protects from necroptosis independent of an impact on cIAP1 or cIAP2, apparently by decreasing recruitment of necrosome components into detergent-resistant complexes.

### Cf inhibits the translocation of the ripoptosome and the necrosome components to detergent-resistant membrane fractions

**Fig. 6:**
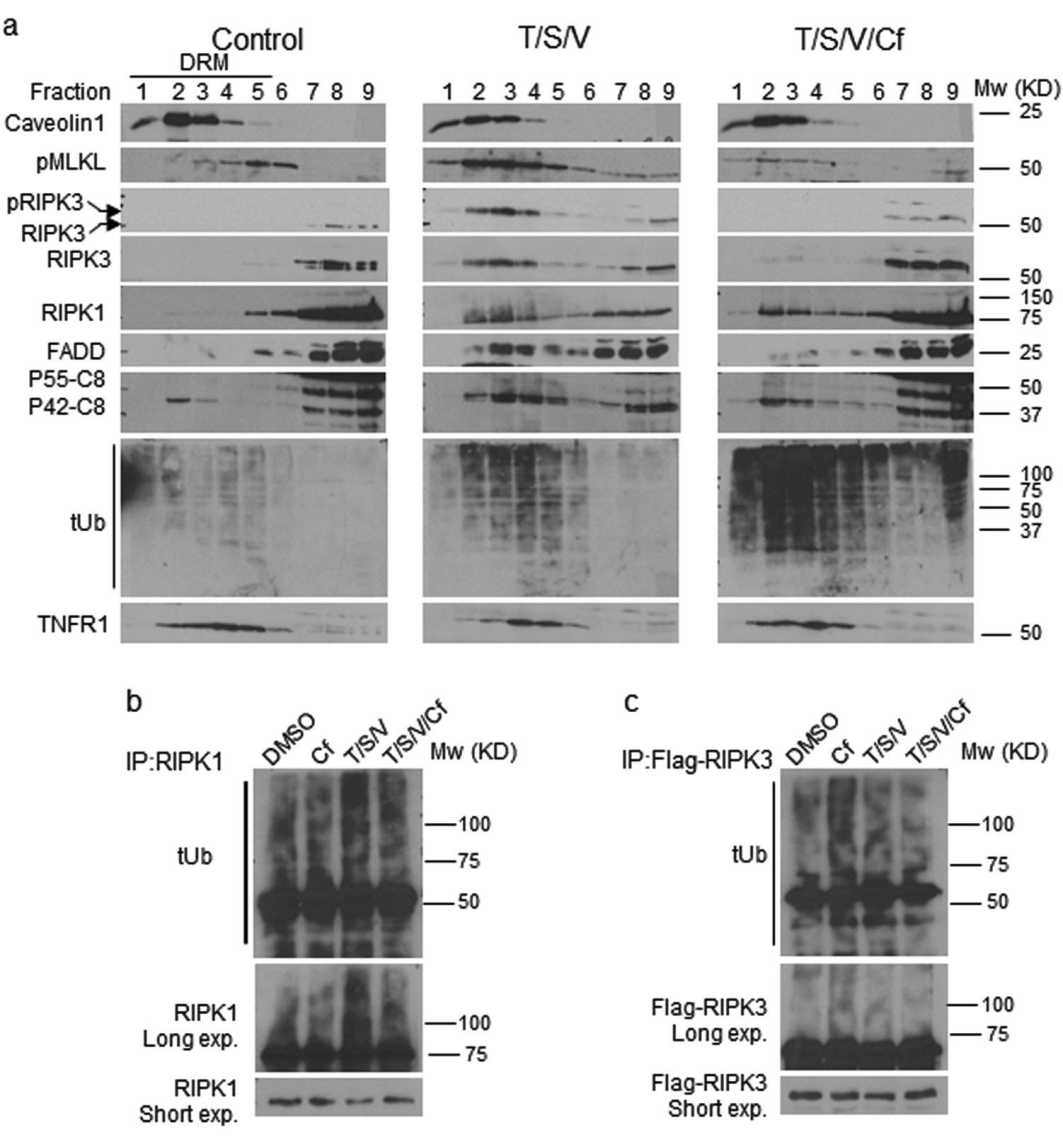
Cf influence on the cell death machinery association with lipid rafts. **a** IB of RIPK1, RIPK3, pRIPK3, FADD, MLKL, TNFR1, and tUb in detergent-resistant membrane fractionations (DRM) of HT-29 cells 6 hpt under the indicated conditions. Caveolin-1 is a marker for DRM. **b** IB of RIPK1 immunoprecipitation (IB/IP) detecting tUb and RIPK1 in HT-29 cell lysates (4 hpt) under reducing conditions. **c** IB of 3xFLAG-tagged RIPK3 IP detecting tUb and FLAG in HT-29 cell lysates (4 hpt) under reducing conditions

The induction of death receptor signaling is associated with translocation of death machinery proteins to detergent-resistant membranes (DRM)^39^. Given that proteasome inhibition appeared to reduce recruitment of necrosome components to detergent-insoluble pellets, we characterized the impact of Cf on translocation of ripotosome and necrosome components to DRMs. Following treatment with T/S/V for 6 h, HT-29 cells showed translocation of death machinery from soluble fractions (7–9) to DRM-containing fractions (1–5; Fig. 6a). Induced pMLKL and pRIPK3, as well as RIPK1, FADD, and P42-Casp8 all showed a similar pattern that extends observations initially made in mouse cells^39^. Total DRM-associated ubiquitinated proteins increased during necroptotic signaling, possibly due to decreased proteasome activity^3^. Moreover, TNFR1 association to DRMs was limited to fractions 4 and 5, following T/S/V. Addition of Cf drove further accumulation of ubiquitinated proteins and reduced pMLKL and pRIPK3 translocation to DRMs, with a modest impact on the pattern of RIPK1, FADD, and P42-Casp8. Overall, these data (Figs. 2c and 6a) align with the predicted activity of RIPK1 upstream of RIPK3 kinase-dependent phosphorylation of MLKL during TNFR1-induced necroptosis^40^. Cf reduced RIPK1 modification, as well as the association of FADD and P42-Casp8 with DRMs, suggesting proteasome activity contributes to DRM-associated ripoptosome composition. Cf also increased levels of TNFR1 in fractions 2 and 3, a pattern similar to control cells. Proteasome inhibition is known to result in the accumulation of K48-ubiquitinated RIPK1 and RIPK3^6,41,42^. Proteasome inhibition for 4 h failed to have the expected impact^40^ and did not alter RIPK1 modification in HT-29 cells (Fig. 6b); however, in agreement with another report^42^, RIPK1 ubiquitination increased during necroptosis. Importantly, Cf reduced this modification, suggesting that some form of RIPK1 ubiquitination may support early steps in necroptosis. In contrast to RIPK1, the modification of RIPK3 in T/S/V/Cf did not appear different from T/S/V (Fig. 6c), even though proteasome inhibition alone appeared to drive increased levels of ubiquitinated RIPK3. These results reveal an unexpected impact of the proteasome^6^ supporting a contribution of RIPK1 ubiquitination to necroptosis in human cells, without altering overall TNFR1 survival signaling.

### Cf inhibits aggregation of ripoptosome and necrosome components

**Fig. 7:**
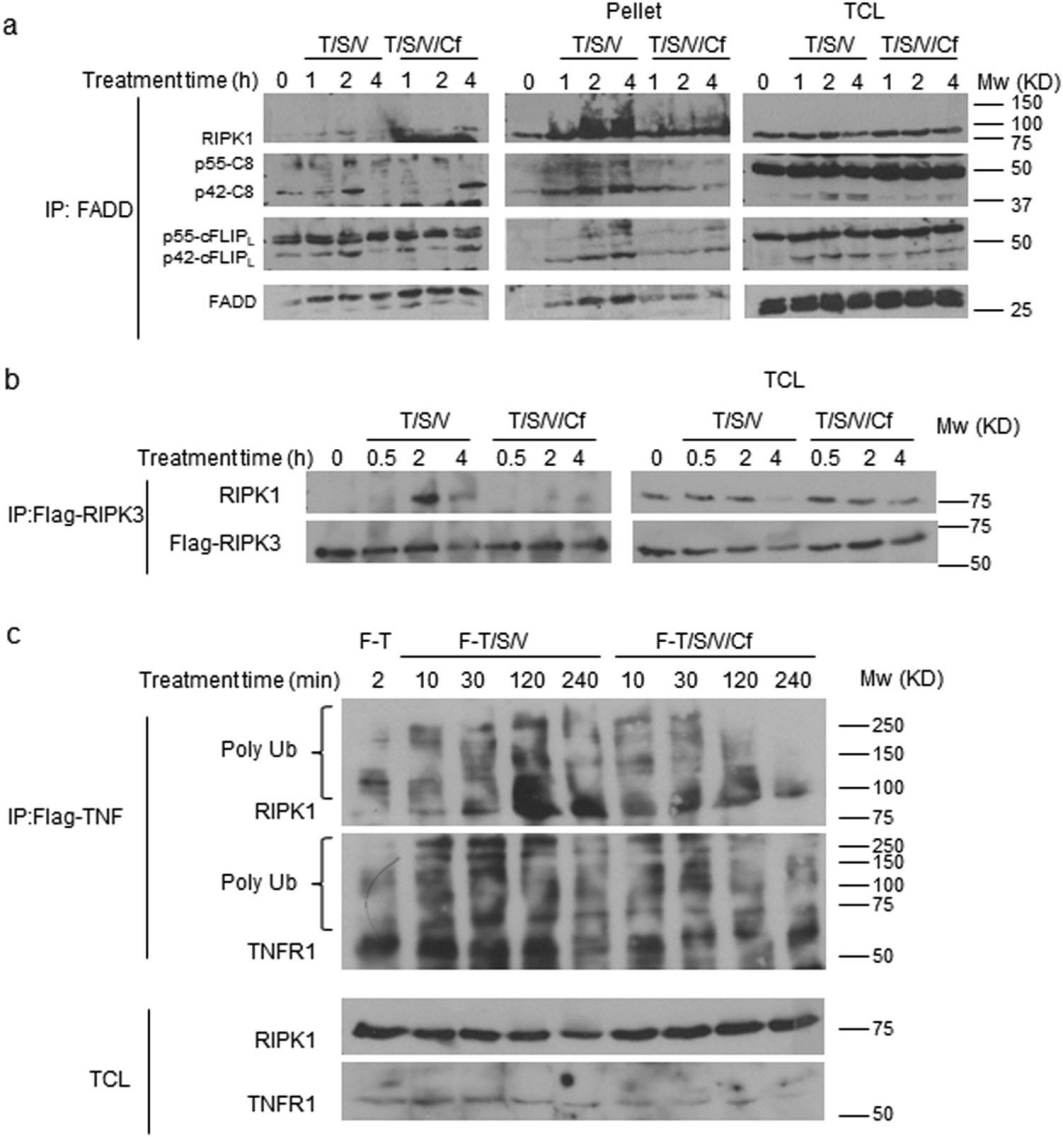
Cf inhibition of ripoptosome and necrosome aggregation. **a**, **b** HT-29 (**a**) or HT-29-FlagRIPK3 (**b**) cell lysates were prepared at the times and treatment conditions indicated for IP/IB detection of the indicated signaling components in Sol. (left panel) compared to pellet fraction (middle panel) and total cell lysates (TCL, right panel). **c** IP/IB detection of HT-29 cell lysates following the treatment with Flag-tagged TNF in combination with S,V, and Cf as designated, for the indicated times

Following TNFR1 activation, RIPK1 interacts with FADD, Casp8 and cFLIP in DRMs that then translocate to form the cytosolic complex IIb (also called a ripoptosome)^40^. RIPK1 recruits RIPK3 to form a necrosome when Casp8 activity is compromised^17,19^. These complexes translocate to the 1% Triton X-100 insoluble fraction (pellet) upon activation of cell death^33,34^. We therefore employed coimmunoprecipitation (co-IP) of FADD^34^ to examine the effect of Cf on the interaction of RIPK1, FADD, cFLIP, and Casp8 in T/S/V-treated HT-29 cells. FADD interacted with RIPK1 within 1 hpt, an interaction that intensified by 2 hpt when additional components of the ripoptosome were present (Fig. 7a, left panel). By 4 hpt, ripoptosome components diminished in the IP-fraction, and appeared in the insoluble pellet (Fig. 7a, middle panel). The interaction of FADD with ripoptosome components was delayed up to 4 h in the presence of Cf where these proteins failed to translocate to the pellet, suggesting a role for proteasome activity in aggregation of this complex^28,34^. Similarly, we analyzed the interaction of RIPK1 with RIPK3, using a stable ectopic expression of 3xFlag-tagged-RIPK3 (Flag-RIPK3) in HT-29 cells^43^. T/S/V drove RIPK1 and RIPK3 interaction within 2 h. This interaction was prevented in the presence of Cf (Fig. 7b). We next evaluated the effect of Cf on RIPK1 interaction with TNFR1 by using Flag-tagged TNF (F-T)^44^. F-T treatment alone for 2 min led to a detectable interaction between modified RIPK1 and modified TNFR1 (Fig. 7c). F-T/S/V increased the interaction and the modifications of RIPK1 and TNFR1 over time. Cf did not affect the early interaction and modification of RIPK1 and TNFR1 (10 and 30 min); however, proteasome inhibition drastically decreased RIPK1 and TNFR1 interaction and modifications at later time points tested (120 and 240 min), effects that could be due to destabilization of RIPK1 interaction with TNFR1. To test this possibility we used NSA to stabilize the necrosome once formed^33^. Cf did not alter this T/S/V/NSA-stabilized necrosome assembly (Fig. 7b, left panel); however, Flag-RIPK3 failed to translocate to the pellet in the presence of Cf. There was also less pellet-associated modified RIPK1 (Fig. 7b, middle panel). Altogether, our results indicate that Cf attenuates TNFR1-induced heavy molecular weight death complex formation and aggregation by destabilizing RIPK1 and TNFR1 interaction and modification.

We further investigated the effect of Cf on ripoptosome and necrosome translocation to the pellet fraction. T/S/V treatment led to phosphorylation and translocation of MLKL 4 hpt, along with modified RIPK1, pRIPK3, and P42-Casp8, hallmarks of ripoptosome and necrosome formation (Supplementary Figure 5b and c). Cf decreased pMLKL levels in association with translocation of unmodified RIPK1, inhibition of pRIPK3 translocation, and poor recruitment of P42-Casp8 to the pellet. As with T/S/V, early T/CH/V signaling was not altered by Cf, but pellet-associated RIPK1 modification, P42-Casp8, cFLIP_L_, and pMLKL levels were reduced (Supplementary Figure 5d and e). Furthermore, MG132 also decreased T/S/V-induced modification and translocation of RIPK1, and was associated with reduced RIPK3 phosphorylation as well as p42-C8 in the pellet (Supplementary Figure 5f). These results suggest that proteasome activity is necessary during necroptosis to support or stabilize the assembly of membrane-associated heavy molecular weight complexes.

## Discussion

Here we found that proteasome function contributes in unexpected ways to execution of necroptosis in human cells. Proteasome inhibitor Cf induced apoptosis in MM cells as well as in cells commonly used to study necroptosis. Moreover, proteasome inhibition dampened necroptotic markers activated downstream of TNFR1 by ameliorating the RIPK1-dependent aggregation of heavy molecular weight death complexes in necroptosis-sensitive human cells.

Evidence has long indicated that proteasome inhibition compromises mitochondrial membrane fidelity to trigger intrinsic apoptosis^3^. In addition, production of mitochondrial reactive oxygen species (ROS)^45^ may contribute to TNFR1-induced necroptosis in some settings^46^. Besides ER stress-associated death, Cf induces extrinsic apoptosis by blocking the degradation of death receptors 4 and 5, and by inducing autocrine secretion of TRAIL ligands^5^, raising the possibility that proteasome inhibition would result in necroptosis when caspases are compromised^47,48^. In agreement with previous publications^3–5,31,49^, Cf induces apoptosis; however, caspase inhibition does not unleash necroptosis even in necroptosis sensitive cells. This aligns with observations showing intrinsic cell death dominates extrinsic death in Cf-treated cells^4^. RIPK3 is rarely preserved in human cancer cell lines and is dispensable for the toxicity of intrinsic cell death inducers, as well as proteasome inhibitors^50^. Intrinsic death does not readily convert to necroptosis when caspase activity is inhibited, even in cells with adequate RIPK3. Notably, RPMI8226 cells had higher Casp8 and RIPK3 levels in comparison to KMS-18 cells; however, these cell lines had equivalent cleavage of Casp3 following Cf treatment, consistent with a mechanism involving intrinsic apoptosis.

Under normal conditions, the proteasome supports TNFR1-complex I survival signaling by degrading IκBα leading to NFκB transactivation^49^. Consistent with this, Cf inhibits the NFκB canonical and non-canonical survival pathways^20,51,52^. In addition, proteasome inhibitors drive cIAP degradation due to mitochondrial release of SMAC/DIABLO^53^, all of which contradicts the pro-survival function of Cf observed in HT-29 cells, and indicates that Cf protection from necroptosis is complex I-independent.

Our results support the observations that SMAC mimetic-induced proteasomal degradation results in accumulation of cIAP2^25,38,50^. The massive accumulation of cIAP2 in necroptosis-sensitive HT-29 after treatment with T/S/V; however, did not influence TNFR1-induced necroptosis, raising a question of whether cIAP2 is protective in this setting. It has long been established that pre-existing cIAP2 protects cancer cells from SMAC mimetic-induced apoptosis^38^, raising the possibility that this protection is dependent on the cell type or the form of death signaling induced.

When CH is employed to sensitize cells, the induction of TNFR1-mediated death relies on proteasomal degradation of cFLIP^22^ and is cIAP-independent. Under these conditions, HT-29 cells do not show a reduction in cFLIP levels even as membrane leakage becomes evident. Furthermore, inhibition of the proteasome activity in the presence of T/CH/V reduces the cleavage and translocation of cFLIP to pellet fractions without obvious alterations in cFLIP levels. T/CH/V cell death is inhibited in *Ripk1*^–/–^ mouse embryonic fibroblasts^54^, but less so in *Mlkl*^*–/–*^ cells^32^, reinforcing our conclusion that Cf attenuates death through altered modification of RIPK1.

Even though proteasome inhibition induces necroptosis in mouse fibroblasts via a mechanism ascribed to polyubiquitinated RIPK3^6^, susceptible human cells accumulate modified RIPK3 without triggering necroptosis. Most cultured human cells^8^, including fibroblasts^43^, do not retain sufficient RIPK3 to support necroptosis. It is not surprising, therefore, that MM cells are relatively insensitive to this pathway. In necroptosis-sensitive HT-29 cells, where the role of RIPK3 was first exposed^8^, proteasome inhibition does not alter ripoptosome assembly but decreases the level of polyubiquitinated RIPK1 associated with pellet and DRM fractions. An analogous reduction in polyubiquitinated RIPK1 occurs with MG132 treatment during TLR3-induced ripoptosome formation^28^. These observations differ from the suggestion that necrosome-associated RIPK1 is deubiquitinated^40^. Ub plays a crucial role in dictating RIPK1-dependent TNFR1 survival signaling such that alterations of RIPK1-associated Ub ligases and deubiquitinases can lead to catastrophic outcomes^19,40^. Our results suggest a role for polyubiquitinated RIPK1 that extends beyond survival signaling such that proteasome activity balances the type of polyubiquitinated species, possibly through distinct RIPK1-Ub linkages or editing proteins such as CYLD and A20^44,55,56^.

In conclusion, our results support a pro-necroptotic function of the proteasome, and provide evidence that a specific proteasome inhibitor compromises necroptosis. Proteasome inhibitors may therefore have adjunct therapeutic value preventing necroptosis-associated inflammatory disorders.

## Materials and methods

### Antibodies and reagents

The following antibodies were from Cell Signaling Technology: anti-Casp8 (9746), anti-Cap3 (9664), anti-cIAP2 (3136), anti-pSAPK/JNK (JNK1/2, 4668), anti-pIκBα (9246), anti-Caveolin1 (3267), and anti-cFLIP (56343). The following antibodies were from Abcam: anti-pMLKL (187091), anti-RIPK3 (72106), anti-pRIPK3 (209384), and anti-FADD (108601). Anti-Ub (S.C8017), and protein A/G-conjugated beads were from Santa Cruz Biotechnology, anti-total MLKL (M6697) was from Sigma-Aldrich, anti-RIPK1 (610459) was from BD Medical Technology, and anti-cIAP1 was a gift from of John Silke (Walter and Eliza Hall Institute of Medical Research). z-VAD-fmk was from Enzo Life Sciences, RIPK1 inhibitor GSK’963, RIPK3 inhibitor GSK’840 and IAP antagonist SMAC007, as well as the pRIP1 S166-specific antibody, were provided by GlaxoSmithKline^34^. IAP antagonist BV6 was provided by Domogoj Vucic (Genentech), recombinant human TNF was from R&D or from PeproTech, Flag-Tagged TNF was from Enzo, necrosulfonamide was from CalBiochem, TLCK (Tosyl-L-lysyl-chloromethane hydrochloride) was from Abcam, cycloheximide (CH) was from Sigma-Aldrich, and Carfilzomib was from BioVision.

### Cells growth and treatments

Human colon cancer cell line HT-29 maintained at 37 °C in 5% CO_2_ using Dulbecco’s modified Eagle’s medium (DMEM) containing 10% FBS (Atlanta Biologicals), 4.5 g/mL glucose, 2 mM l-glutamine, 100 U/mL penicillin and 100 U/mL streptomycin (Invitrogen). The MM cell lines RPMI8226, MM1.s and KMS-18 were provided by Lawrence Boise (Emory University), and were maintained in complete RPMI medium containing 10% FBS, 2 mM l-glutamine, 100 U/mL penicillin, and 100 U/mL streptomycin. For the induction of necroptosis, cells were treated with T/BV6/V (30 ηg/mL, 25 µM, and 0.1 µM BV6, respectively) or with T/SMA007/V (1 µM SMA007), or with T/CH/V (50 µg/mL cycloheximide) as indicated. Cells were either treated for 22 h for viability assays, or for 8 h (MM) and 6 h (HT-29), for IB assays. GSK'840, GSK'963, and NSA, were dissolved in DMSO as supplier recommended^34^, and were used as indicated in the text to inhibit necroptosis. Cf was dissolved in DMSO to a stock concentration of 5 mM as supplier indicated, and were used in 100 ηM concentration to induce death in MM cells, and 1 µM in HT-29 cells. DMSO dissolved in medium was used as treatment control. For TNFR1 immunoprecipitation, cells were treated with 100 ηg/mL flag-tagged TNF, either alone or in combination with SMA007 and V and Cf as indicated.

### Plasmids and lentivirus stable transduction

Human-3XFlag-RIPK3 expression vector was previously described^43^. Briefly, hRIPK3 open reading frame (ORF) was inserted into pLV-EF1α-MCS-IRES-Puro lentiviral vector (Biosettia). Three-tandem FLAG epitope-tagged hRIP3 expression plasmid was constructed by inserting hRIPK3 ORF into p3xFLAG-CMV10 vector (Sigma). cIAP1 and cIAP2 knockdown vectors pLKO-shcIAP1a (TRC0000003780), pLKO-shcIAP1b (TRC0000003782), pLKO-shcIAP2a (TRC0000003778), and pLKO-shcIAP2b (TRC0000003776), from Adgene, were previously described^57^. Transient transfections were performed with Lipofectamine LTX with Plus reagent (Invitrogen). Lentivirus stock was prepared from 293T cells that were transfected with pLV-hRIPK3 or pLKO.1 constructs along with psPAX2 and VSV-G-expressing plasmids. Low passage HT-29 cells were transduced with lentiviral vector and selected with 2 µg/mL puromycin (Invitrogen).

### Immunoprecipitation and Immunoblots

Whole-cell extracts were prepared using lysis buffer [50 mM Tris, 150 mM NaCl, 5 mM EDTA, 1% Triton X-100, including phosphatase and protease inhibitors (Sigma-Aldrich)], and clarified cell lysates were incubated over-night with anti-FADD or anti-Flag, mixed with protein A/G agarose beads (Santa Cruz). For immunoblots, samples were resolved in 10% SDS-Polyacrylamide gels; proteins were transferred to Immobilon PVDF membrane (Millipore) and developed using specified Abs. Alternatively, whole cell lysates were centrifuged at 15000 RPM (20 min, 4 °C) for the separation of 1% Triton X-100 soluble (sol.), and insoluble (pellet) fractions^34^.

### Cell fractionation

DRM fractions were prepared as previously described^58^ with the following modifications. Cells were grown in 182 cm^2^ tissue culture flasks, up to 80% confluency, treated with T/S/V or T/S/V/Cf for 6 h, and then scraped and washed by centrifugation with cold PBS. Cell pellets were lysed in a Dounce homogenizer with DRMs lysis buffer (0.1% Triton X-100, 100 mM NaCl, 2 mM EDTA, 2 mM EGTA, 30 mM HEPES, pH 7.5, 1 mM Na_3_VO_4_, 50 μM phenylarsine oxide, protease and phosphatase inhibitors). Homogenates were centrifuged at 400xgav (3 min, 4 °C). Optiprep and sucrose were added to the supernatant to a final concentration of 40% Optiprep and 10% sucrose, which was overlaid with 35, 30, 25, 20 and 0% Optiprep and 10% sucrose and centrifuged (6 h, 170,000xgav, 4 °C). Nine fractions were collected from the top of the gradient.

### Cell viability

Cells were incubated 18–22 h, as indicated, and then viability was assessed using Cell Titer-Glo Luminescent Cell Viability Assay (Promega)^14^. Values depict viability as a percentage of DMSO treated cells. Alternatively, cells were cultured with 62.5 nM SYTOX Green (Invitrogen), a live-cell impermeant nucleic acid fluorescent dye, and analyzed by an IncuCyte ZOOM live-cell imaging and analysis system (Essen Biosystems). Green cells per square millimeter were calculated from four images at indicated points, and values depict mortality (membrane permeability) as a percent of T/CH or T/S/V treated cells, as indicated.

### Statistical analyses

Statistical comparisons employed parametric evaluation using Student’s *t* test (GraphPad Prism software, or Microsoft Excel). All experiments were repeated at least three times with similar results, and data are represented as the mean ± S.D.

## Electronic supplementary material

Figure s1

Figure s2

Figure s3

Figure s4

Figure s5

Supplementary figure Legends
