# Supplementary figures and images for: Proteasome inhibition blocks necroptosis by attenuating death complex aggregation
Source: Cell Death Dis. 2018 Mar 1;9(3):346. doi: 10.1038/s41419-018-0371-x (PMC5832869; doi:10.1038/s41419-018-0371-x)

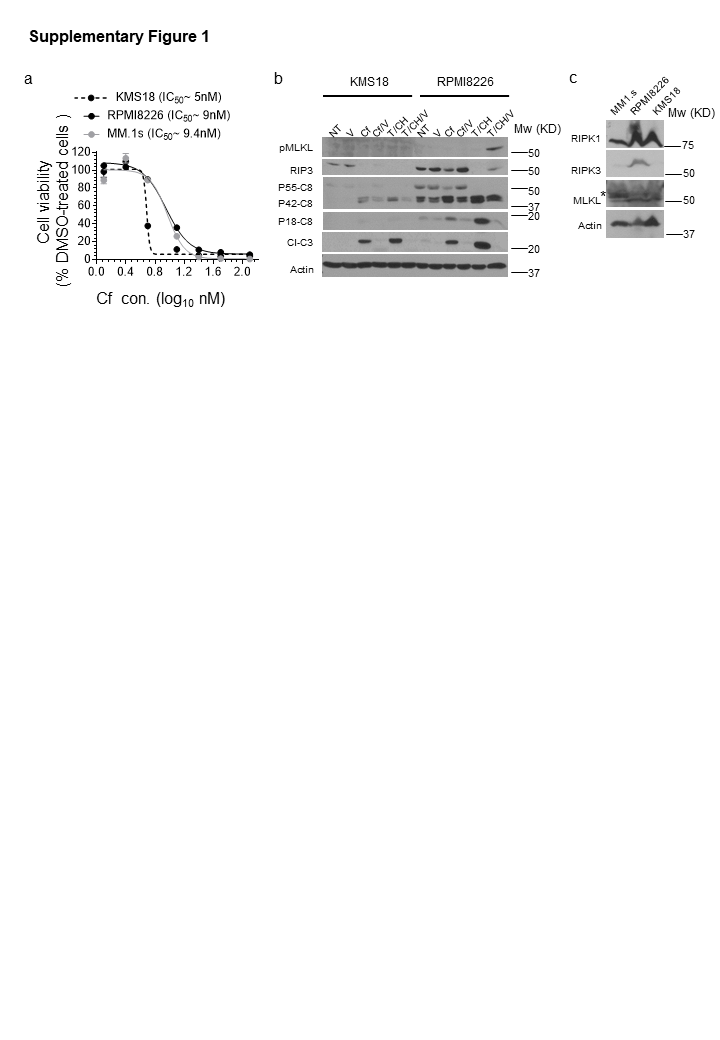

Supplement: Supplementary file 1 — Figure s1 [file 41419_2018_371_MOESM1_ESM.tif]

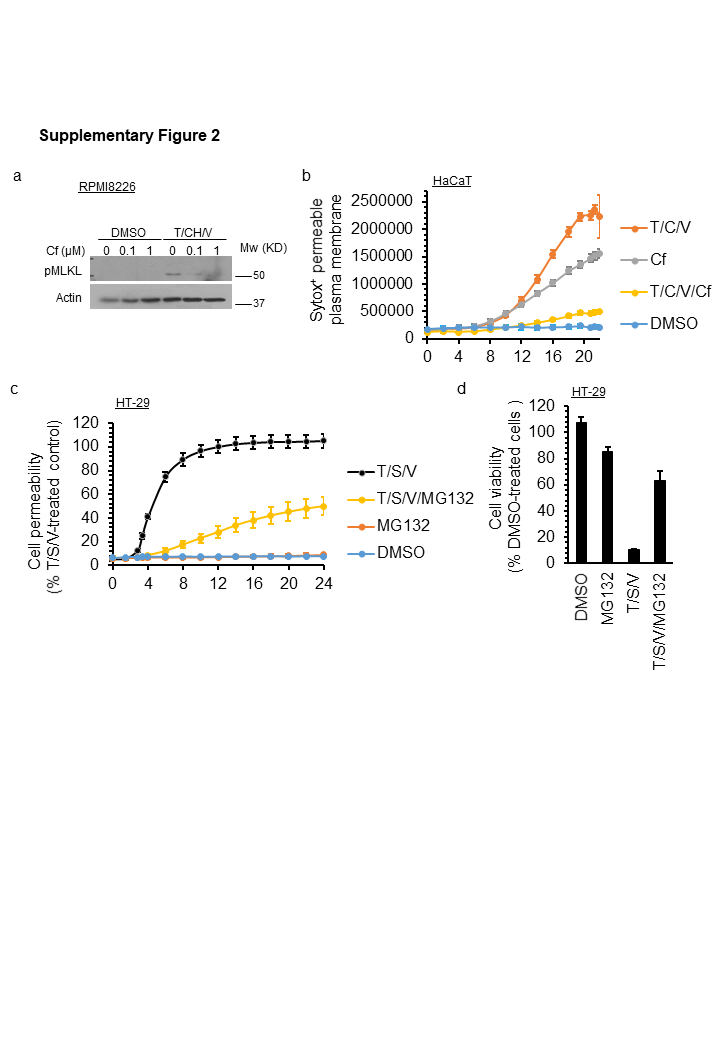

Supplement: Supplementary file 2 — Figure s2 [file 41419_2018_371_MOESM2_ESM.tif]

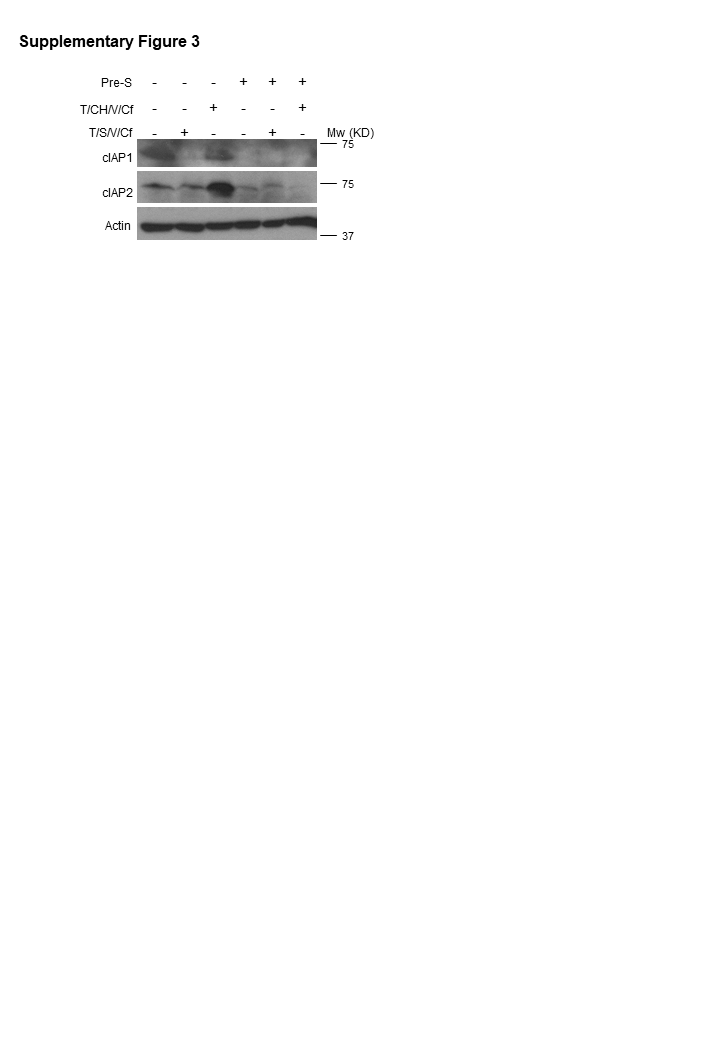

Supplement: Supplementary file 3 — Figure s3 [file 41419_2018_371_MOESM3_ESM.tif]

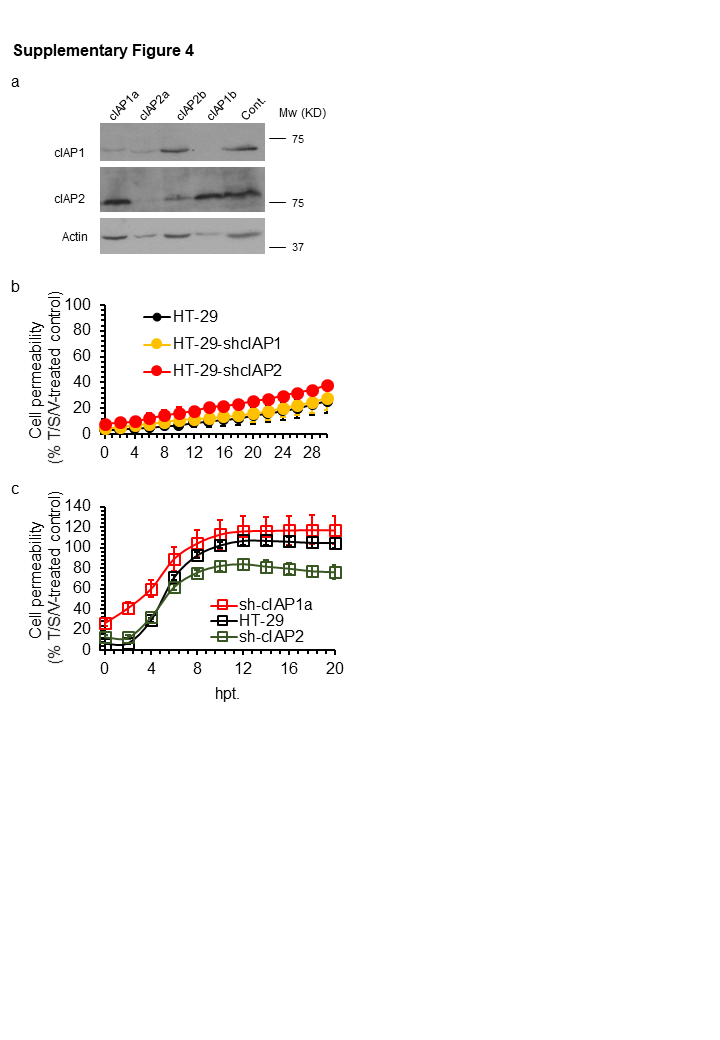

Supplement: Supplementary file 4 — Figure s4 [file 41419_2018_371_MOESM4_ESM.tif]

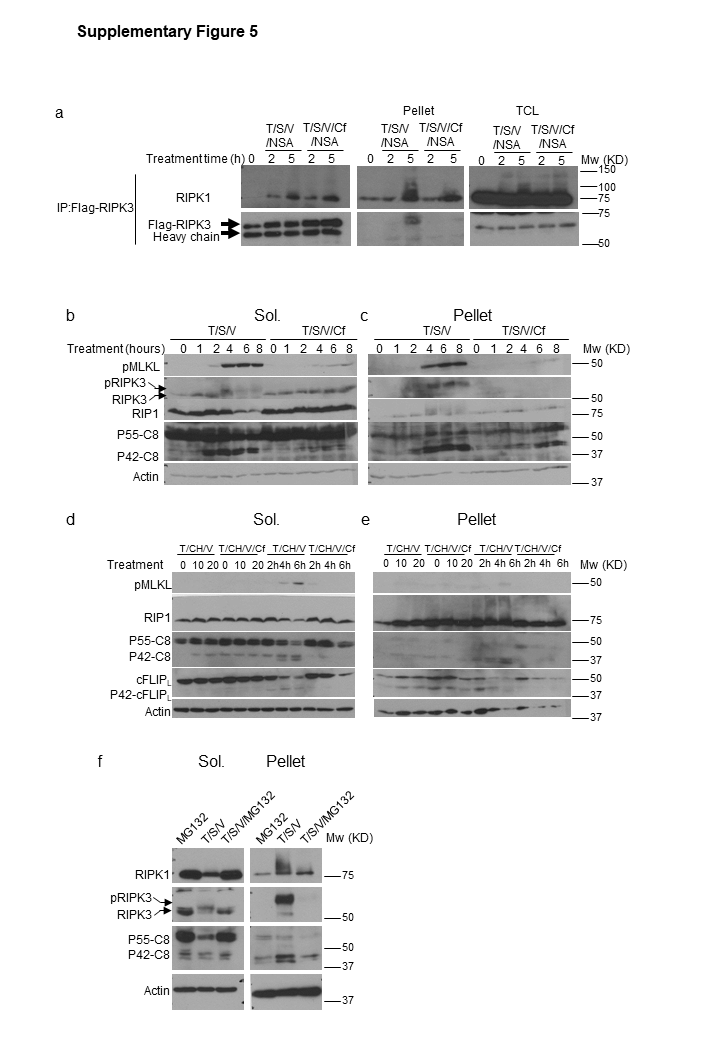

Supplement: Supplementary file 5 — Figure s5 [file 41419_2018_371_MOESM5_ESM.tif]
