# Supplementary material for: Proteasome inhibition blocks necroptosis by attenuating death complex aggregation
Source: Cell Death Dis. 2018 Mar 1;9(3):346. doi: 10.1038/s41419-018-0371-x (PMC5832869; doi:10.1038/s41419-018-0371-x)
Supplement: Supplementary file 6 — Supplementary figure Legends [file 41419_2018_371_MOESM6_ESM.docx]

**Supplementary figure Legends**

**Supplementary figure 1. Involvement of apoptosis in Cf mediated death in MM cells.** (a) Viability measurement of MM cell lines following 24 h of the indicated Cf treatments (b) IB of pMLKL, RIPK3, Casp8 cleavage products, and cleaved Casp3 in MM cells treated with Cf, or Cf/V or T/CH, or T/CH/V for 8 hours. (c) IB of RIPK1, RIPK3, and MLKL (with asterisk indicating cross-reactive bands) in MM cells.

**Supplementary figure 2.** **Cf inhibition of necroptosis in RPMI8226 MM cells.** (a) IB of pMLKL in RPMI8226 cells following 8 h treatment with the indicated concentrations of Cf alone or with T/CH/V. (b) Time course of HaCaT cell Sytox^+^ membrane permeable, following treatment with Cf alone, T/CH/V, or T/CH/V/Cf. (c) Time course of HT-29 cell membrane permeability following treatment with MG132, T/S/V or T/S/V/Cf, depicted as a percentage of T/CH-treated (24 h) control. (d) Viability of HT-29 cells 24 hpt under the indicated conditions.

**Supplementary figure 3.** **Cf effect on cIAP levels in S-pretreated HT-29 cells.** IB of cIAP1 and cIAP2 in HT-29 cells either pretreated with DMSO or S for 30 min, followed by the indicated treatments.

**Supplementary figure 4. Effect of cIAP1 and cIAP2 knock down on HT-29 response to Cf or TNF induced necroptosis.** (a) IB of cIAP1 and cIAP2 in HT-29 cells stably expressing shRNA against cIAP1 (cIAP1a and cIAP1b), or cIAP2 (cIAP2a and cIAP2b). (b and c) Time course of cell membrane permeability of HT-29-shcIAP1a and shcIAP2b and parental HT-29 cells, following treatment with Cf (b) or T/S/V (c) as indicated, depicted as a percentage of T/S/V treated (24 hpt) HT-29 parental cells.

**Supplementary figure 5.** **Cf attenuation of the translocation of the ripoptosome and necrosome components to the pellet fraction.** (a) HT-29-FlagRIPK3 cell lysates were prepared at the times and treatment conditions indicated for IP/IB detection of the indicated signaling components in Sol. (left panel) compared to Pellet fraction (middle panel) and total cell lysates (TCL, right panel). (b to e) IB of pMLKL, pRIPK3, RIPK1, Casp8, and cFLIP in Sol. and Pellet fractions, following T/S/V treatment (a and b), or T/CH/V treatment (c and d), for the indicated times.
